# *N*-(4-Bromo-2-methyl­phen­yl)pivalamide

**DOI:** 10.1107/S1600536809034345

**Published:** 2009-09-05

**Authors:** Wei-Xia Qing, Wei Zhang

**Affiliations:** a Medical College of Henan University, Henan University, Kaifeng 475004, People’s Republic of China; b Department of Pharmacy, Zhengzhou Railway Vocational and Technological College, Zhengzhou 450052, People’s Republic of China

## Abstract

The conformation of the N—H bond in the title compound, C_12_H_16_BrNO, is *syn* to the *ortho*-methyl substituent. There are two unique molecules in the asymmetric unit. In the crystal structure, inter­molecular N—H⋯O hydrogen bonds link the mol­ecules, forming infinite chains down [010].

## Related literature

For a study of the effect of ring and side-chain substitution on the crystal structures of aromatic amides, see: Gowda *et al.* (2007 ▶). For related structures, see: Gowda *et al.* (2007*a* ▶,*b* ▶,*c* ▶).
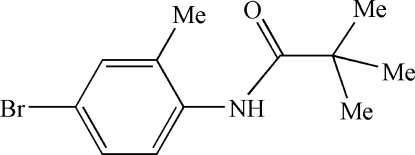


## Experimental

### 

#### Crystal data

C_12_H_16_BrNO
*M* *_r_* = 270.17
Monoclinic, 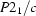

*a* = 11.764 (3) Å
*b* = 19.584 (5) Å
*c* = 12.956 (3) Å
β = 117.877 (19)°
*V* = 2638.5 (11) Å^3^
*Z* = 8
Mo *K*α radiation
μ = 3.09 mm^−1^
*T* = 293 K
0.42 × 0.37 × 0.32 mm

#### Data collection

Bruker SMART CCD area-detector diffractometer
Absorption correction: multi-scan (*SADABS*; Sheldrick, 2001 ▶) *T*_min_ = 0.357, *T*_max_ = 0.438
24481 measured reflections
4634 independent reflections
1875 reflections with *I* > 2σ(*I*)
*R*_int_ = 0.113

#### Refinement

*R*[*F* ^2^ > 2σ(*F* ^2^)] = 0.064
*wR*(*F* ^2^) = 0.211
*S* = 1.01
4634 reflections
271 parameters
65 restraints
H-atom parameters constrained
Δρ_max_ = 0.90 e Å^−3^
Δρ_min_ = −0.70 e Å^−3^

### 

Data collection: *SMART* (Bruker, 2001 ▶); cell refinement: *SAINT-Plus* (Bruker, 2001 ▶); data reduction: *SAINT-Plus*; program(s) used to solve structure: *SHELXS97* (Sheldrick, 2008 ▶); program(s) used to refine structure: *SHELXL97* (Sheldrick, 2008 ▶); molecular graphics: *PLATON* (Spek, 2009 ▶); software used to prepare material for publication: *PLATON*.

## Supplementary Material

Crystal structure: contains datablocks global, I. DOI: 10.1107/S1600536809034345/at2861sup1.cif

Structure factors: contains datablocks I. DOI: 10.1107/S1600536809034345/at2861Isup2.hkl

Additional supplementary materials:  crystallographic information; 3D view; checkCIF report

## Figures and Tables

**Table 1:** Hydrogen-bond geometry (Å, °)

*D*—H⋯*A*	*D*—H	H⋯*A*	*D*⋯*A*	*D*—H⋯*A*
N1—H1*A*⋯O2^i^	0.86	2.14	2.989 (8)	170
N2—H2*B*⋯O1^ii^	0.86	2.14	2.943 (8)	155

Symmetry codes: (i) 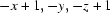; (ii) 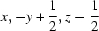.

**Fig. 1.**
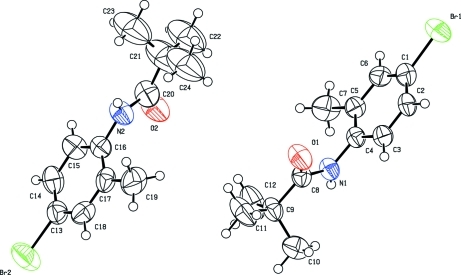
The molecular structure of (I), showing the atom-labelling scheme. Displacement ellipsoids are drawn at the 50% probability level.

**Fig. 2.**
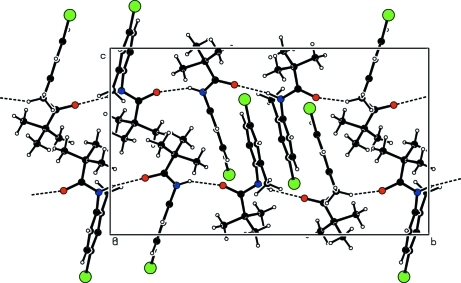
One-dimensional structure of (I) along [010] direction, Hydrogen bonds are shown in the dashing line.

## supplementary crystallographic information

### Comment

As part of a study of the effect of ring and side chain substitutions on the crystal structures of chemically and biologically important class of compounds such as aromatic amides (Gowda, Kozisek *et al.*, 2007), We now report the the crystal structure of the title compound, (I).

As shown in Fig.1, the title compound includes both the *ortho*-methyl and the *p*-Br-substituted phenyl group and an imide group. The title compound, (I), (Fig. 1) is structural isomer of both the 2-chloro and the 3-chloro substituent in *N*-(2,3-dichlorophenyl)acetamide (Gowda *et al.*,  2007*a*) and *N*-(2,3-Dichlorophenyl)-2,2,2-trimethylacetamide (Gowda *et al.*,  2007*b*). The conformation of the N–H bond in the title compound is *syn* to the *ortho*-methyl substituent, similar to that in both the 2-chloro and the 3-chloro-substituted amides, but in contrast to the *anti* conformation observed for the corresponding 3-chloro-substituted *N*-(3-Chlorophenyl)-2,2,2-trimethylacetamide (Gowda *et al.*,  2007*c*). The amide H atom is involved in an intramolecular hydrogen bond with the O atom of the carbonyl group.

In the crystal structure, these molecules are linked into infinite one-dimensional chains by intermolecular N–H···O hydrogen bonds running along [010] direction (Fig. 2, Table 1).

### Experimental

2,2,2-Trimethyl-*N*-(2-methylphenyl)acetamide (0.95 5 g, 5 mmol) was added slowly by cannulation to a stirred suspension of *p*-nitroaniline (0.690 g, 3 mmol) in chloroform (50 ml) at room temperature. After stirring for 2 h the solution was quenched with saturated aqueous sodium bicarbonate solution (20 ml) the layers were separated and the aqueous layer was extracted with chloroform, the combined organic extracts were washed with water (20 ml), dried (MgSO_4_) and evaporated under reduced pressure to give the crude product as viscous brown oil. Then purification by short column chromatography (chloroform) and recrystallization from chloroform gave the compound (I) as brown needles crystals (1.094 g, 81%).

### Refinement

H atoms were treated as riding, with C—H distances in the range of 0.93–0.96 Å and N—H distances of 0.86 Å, and were refined as riding with *U*_iso_(*H*) = 1.2*U*_eq_(N and C in phenyl ring) and *U*_iso_(*H*) = 1.5*U*_eq_(C_methyl_).

### Crystal data

**Table:**

C_12_H_16_BrNO	*F*(000) = 1104
*M**_r_* = 270.17	*D*_x_ = 1.360 Mg m^−^^3^
Monoclinic, *P*2_1_/*c*	Mo *K*α radiation, λ = 0.71073 Å
Hall symbol: -P 2ybc	Cell parameters from 3412 reflections
*a* = 11.764 (3) Å	θ = 2.2–19.4°
*b* = 19.584 (5) Å	µ = 3.09 mm^−^^1^
*c* = 12.956 (3) Å	*T* = 293 K
β = 117.877 (19)°	Block, colourless
*V* = 2638.5 (11) Å^3^	0.42 × 0.37 × 0.32 mm
*Z* = 8	

### Data collection

**Table:**

Bruker SMART CCD area-detector diffractometer	4634 independent reflections
Radiation source: fine-focus sealed tube	1875 reflections with *I* > 2σ(*I*)
graphite	*R*_int_ = 0.113
φ and ω scans	θ_max_ = 25.0°, θ_min_ = 2.0°
Absorption correction: multi-scan (*SADABS*; Sheldrick, 2001)	*h* = −13→13
*T*_min_ = 0.357, *T*_max_ = 0.438	*k* = −23→23
24481 measured reflections	*l* = −15→15

### Refinement

**Table:**

Refinement on *F*^2^	Primary atom site location: structure-invariant direct methods
Least-squares matrix: full	Secondary atom site location: difference Fourier map
*R*[*F*^2^ > 2σ(*F*^2^)] = 0.064	Hydrogen site location: inferred from neighbouring sites
*wR*(*F*^2^) = 0.211	H-atom parameters constrained
*S* = 1.00	*w* = 1/[σ^2^(*F*_o_^2^) + (0.0858*P*)^2^ + 4.1364*P*] where *P* = (*F*_o_^2^ + 2*F*_c_^2^)/3
4634 reflections	(Δ/σ)_max_ = 0.001
271 parameters	Δρ_max_ = 0.90 e Å^−^^3^
65 restraints	Δρ_min_ = −0.70 e Å^−^^3^

### Special details

**Table:**

Geometry. All e.s.d.'s (except the e.s.d. in the dihedral angle between two l.s. planes) are estimated using the full covariance matrix. The cell e.s.d.'s are taken into account individually in the estimation of e.s.d.'s in distances, angles and torsion angles; correlations between e.s.d.'s in cell parameters are only used when they are defined by crystal symmetry. An approximate (isotropic) treatment of cell e.s.d.'s is used for estimating e.s.d.'s involving l.s. planes.
Refinement. Refinement of *F*^2^ against ALL reflections. The weighted *R*-factor *wR* and goodness of fit *S* are based on *F*^2^, conventional *R*-factors *R* are based on *F*, with *F* set to zero for negative *F*^2^. The threshold expression of *F*^2^ > σ(*F*^2^) is used only for calculating *R*-factors(gt) *etc*. and is not relevant to the choice of reflections for refinement. *R*-factors based on *F*^2^ are statistically about twice as large as those based on *F*, and *R*- factors based on ALL data will be even larger.

### Fractional atomic coordinates and isotropic or equivalent isotropic displacement parameters (Å^2^)

**Table:**

	*x*	*y*	*z*	*U*_iso_*/*U*_eq_	
Br1	0.96107 (9)	0.07766 (6)	1.22541 (8)	0.0941 (5)	
Br2	0.00476 (11)	0.12629 (7)	−0.17802 (9)	0.1081 (5)	
N1	0.4710 (6)	0.0356 (3)	0.7724 (5)	0.0521 (16)	
H1A	0.4492	−0.0041	0.7412	0.063*	
N2	0.4775 (6)	0.2083 (3)	0.2649 (5)	0.0665 (19)	
H2B	0.4777	0.2497	0.2871	0.080*	
O1	0.4160 (5)	0.1460 (3)	0.7638 (5)	0.0731 (17)	
O2	0.5853 (6)	0.1099 (3)	0.3045 (5)	0.089 (2)	
C1	0.8081 (8)	0.0605 (4)	1.0861 (7)	0.058 (2)	
C2	0.6924 (8)	0.0724 (4)	1.0821 (6)	0.054 (2)	
H2A	0.6884	0.0871	1.1486	0.065*	
C3	0.5816 (8)	0.0626 (4)	0.9801 (6)	0.053 (2)	
H3A	0.5024	0.0697	0.9780	0.064*	
C4	0.5866 (7)	0.0423 (3)	0.8792 (6)	0.0465 (19)	
C5	0.7052 (8)	0.0280 (4)	0.8833 (6)	0.052 (2)	
C6	0.8141 (8)	0.0384 (4)	0.9878 (7)	0.060 (2)	
H6A	0.8942	0.0303	0.9922	0.073*	
C7	0.7118 (8)	0.0065 (5)	0.7749 (7)	0.081 (3)	
H7A	0.7998	−0.0013	0.7934	0.121*	
H7B	0.6764	0.0419	0.7171	0.121*	
H7C	0.6634	−0.0348	0.7450	0.121*	
C8	0.3938 (7)	0.0895 (4)	0.7178 (6)	0.0503 (18)	
C9	0.2783 (8)	0.0769 (4)	0.5992 (6)	0.0594 (19)	
C10	0.1890 (9)	0.0268 (5)	0.6154 (8)	0.094 (3)	
H10A	0.1604	0.0462	0.6674	0.142*	
H10B	0.2341	−0.0150	0.6478	0.142*	
H10C	0.1159	0.0178	0.5412	0.142*	
C11	0.2083 (9)	0.1434 (4)	0.5504 (8)	0.098 (3)	
H11A	0.1838	0.1633	0.6048	0.147*	
H11B	0.1328	0.1349	0.4777	0.147*	
H11C	0.2638	0.1743	0.5376	0.147*	
C12	0.3226 (10)	0.0449 (5)	0.5156 (7)	0.100 (3)	
H12A	0.3794	0.0759	0.5047	0.150*	
H12B	0.2491	0.0360	0.4416	0.150*	
H12C	0.3670	0.0029	0.5481	0.150*	
C13	0.1525 (9)	0.1490 (4)	−0.0371 (7)	0.062 (2)	
C14	0.2695 (10)	0.1463 (4)	−0.0340 (7)	0.070 (2)	
H14A	0.2766	0.1318	−0.0991	0.084*	
C15	0.3774 (8)	0.1652 (4)	0.0661 (7)	0.067 (2)	
H15A	0.4576	0.1640	0.0684	0.080*	
C16	0.3671 (8)	0.1861 (4)	0.1639 (7)	0.053 (2)	
C17	0.2495 (10)	0.1873 (4)	0.1621 (7)	0.064 (2)	
C18	0.1419 (9)	0.1689 (4)	0.0592 (8)	0.068 (2)	
H18A	0.0612	0.1701	0.0558	0.081*	
C19	0.2349 (10)	0.2096 (5)	0.2664 (7)	0.093 (3)	
H19A	0.1461	0.2068	0.2483	0.139*	
H19B	0.2643	0.2558	0.2860	0.139*	
H19C	0.2851	0.1803	0.3314	0.139*	
C20	0.5824 (9)	0.1700 (5)	0.3295 (8)	0.074 (2)	
C21	0.6956 (13)	0.2023 (7)	0.4300 (12)	0.149 (3)	
C22	0.8063 (12)	0.1606 (6)	0.4813 (11)	0.152 (3)	
H22A	0.7860	0.1187	0.5075	0.228*	
H22B	0.8731	0.1840	0.5466	0.228*	
H22C	0.8350	0.1507	0.4247	0.228*	
C23	0.7298 (12)	0.2717 (6)	0.3915 (11)	0.152 (3)	
H23A	0.8004	0.2926	0.4573	0.228*	
H23B	0.6565	0.3015	0.3628	0.228*	
H23C	0.7534	0.2637	0.3310	0.228*	
C24	0.6485 (12)	0.2294 (6)	0.5109 (11)	0.154 (3)	
H24A	0.6243	0.1921	0.5445	0.230*	
H24B	0.5752	0.2583	0.4683	0.230*	
H24C	0.7155	0.2553	0.5719	0.230*	

### Atomic displacement parameters (Å^2^)

**Table:**

	*U*^11^	*U*^22^	*U*^33^	*U*^12^	*U*^13^	*U*^23^
Br1	0.0669 (7)	0.1288 (10)	0.0607 (6)	−0.0185 (6)	0.0082 (5)	−0.0030 (6)
Br2	0.0852 (8)	0.1378 (11)	0.0688 (7)	−0.0078 (7)	0.0089 (6)	−0.0144 (7)
N1	0.059 (4)	0.042 (4)	0.045 (4)	0.005 (3)	0.016 (3)	−0.001 (3)
N2	0.073 (5)	0.048 (4)	0.059 (4)	0.004 (4)	0.014 (4)	−0.010 (4)
O1	0.082 (4)	0.042 (3)	0.064 (3)	0.003 (3)	0.008 (3)	−0.005 (3)
O2	0.096 (5)	0.045 (4)	0.084 (4)	0.010 (3)	0.006 (4)	−0.018 (3)
C1	0.062 (6)	0.056 (5)	0.053 (5)	0.006 (4)	0.024 (4)	0.005 (4)
C2	0.063 (6)	0.059 (5)	0.037 (4)	0.003 (4)	0.021 (4)	0.000 (4)
C3	0.055 (5)	0.055 (5)	0.048 (5)	0.001 (4)	0.024 (4)	−0.001 (4)
C4	0.053 (5)	0.037 (4)	0.043 (5)	0.003 (4)	0.017 (4)	0.001 (4)
C5	0.056 (6)	0.053 (5)	0.049 (5)	0.000 (4)	0.026 (4)	−0.004 (4)
C6	0.047 (5)	0.068 (6)	0.066 (6)	0.002 (4)	0.026 (5)	0.008 (5)
C7	0.078 (7)	0.100 (7)	0.083 (6)	−0.002 (5)	0.052 (6)	−0.023 (6)
C8	0.059 (4)	0.047 (5)	0.044 (4)	0.000 (4)	0.023 (3)	−0.003 (4)
C9	0.069 (5)	0.049 (4)	0.044 (4)	0.001 (3)	0.013 (3)	−0.002 (3)
C10	0.078 (6)	0.100 (7)	0.081 (6)	−0.023 (5)	0.016 (5)	−0.001 (5)
C11	0.093 (7)	0.069 (5)	0.072 (6)	0.015 (5)	−0.010 (5)	0.001 (4)
C12	0.120 (8)	0.120 (7)	0.050 (5)	0.026 (6)	0.031 (5)	−0.012 (5)
C13	0.072 (7)	0.060 (6)	0.050 (5)	−0.003 (5)	0.026 (5)	0.000 (4)
C14	0.089 (7)	0.071 (6)	0.048 (5)	0.011 (5)	0.031 (5)	−0.004 (4)
C15	0.066 (6)	0.070 (6)	0.063 (6)	0.007 (5)	0.029 (5)	−0.011 (5)
C16	0.057 (6)	0.038 (5)	0.057 (5)	0.006 (4)	0.020 (5)	0.001 (4)
C17	0.095 (7)	0.049 (5)	0.048 (5)	−0.012 (5)	0.033 (5)	−0.003 (4)
C18	0.074 (6)	0.069 (6)	0.074 (6)	−0.010 (5)	0.045 (6)	−0.002 (5)
C19	0.118 (8)	0.112 (8)	0.069 (6)	−0.025 (7)	0.061 (6)	−0.019 (6)
C20	0.087 (6)	0.054 (5)	0.072 (5)	0.005 (5)	0.029 (5)	−0.004 (4)
C21	0.125 (5)	0.105 (5)	0.136 (5)	−0.001 (4)	−0.006 (4)	−0.032 (4)
C22	0.126 (6)	0.106 (5)	0.138 (6)	0.000 (4)	−0.010 (4)	−0.032 (4)
C23	0.126 (6)	0.108 (5)	0.140 (6)	−0.004 (4)	−0.007 (4)	−0.029 (4)
C24	0.129 (6)	0.112 (5)	0.137 (6)	−0.002 (4)	−0.006 (4)	−0.032 (4)

### Geometric parameters (Å, °)

**Table:**

Br1—C1	1.889 (8)	C11—H11B	0.9600
Br2—C13	1.894 (8)	C11—H11C	0.9600
N1—C8	1.355 (9)	C12—H12A	0.9600
N1—C4	1.422 (9)	C12—H12B	0.9600
N1—H1A	0.8600	C12—H12C	0.9600
N2—C20	1.349 (10)	C13—C14	1.359 (11)
N2—C16	1.413 (9)	C13—C18	1.368 (11)
N2—H2B	0.8600	C14—C15	1.375 (11)
O1—C8	1.225 (8)	C14—H14A	0.9300
O2—C20	1.226 (9)	C15—C16	1.389 (11)
C1—C2	1.358 (10)	C15—H15A	0.9300
C1—C6	1.377 (11)	C16—C17	1.374 (11)
C2—C3	1.368 (10)	C17—C18	1.390 (11)
C2—H2A	0.9300	C17—C19	1.503 (11)
C3—C4	1.393 (10)	C18—H18A	0.9300
C3—H3A	0.9300	C19—H19A	0.9600
C4—C5	1.399 (10)	C19—H19B	0.9600
C5—C6	1.377 (10)	C19—H19C	0.9600
C5—C7	1.503 (10)	C20—C21	1.499 (14)
C6—H6A	0.9300	C21—C22	1.412 (15)
C7—H7A	0.9600	C21—C24	1.494 (18)
C7—H7B	0.9600	C21—C23	1.564 (17)
C7—H7C	0.9600	C22—H22A	0.9600
C8—C9	1.521 (10)	C22—H22B	0.9600
C9—C11	1.513 (10)	C22—H22C	0.9600
C9—C10	1.522 (11)	C23—H23A	0.9600
C9—C12	1.539 (11)	C23—H23B	0.9600
C10—H10A	0.9600	C23—H23C	0.9600
C10—H10B	0.9600	C24—H24A	0.9600
C10—H10C	0.9600	C24—H24B	0.9600
C11—H11A	0.9600	C24—H24C	0.9600
			
C8—N1—C4	122.7 (6)	C9—C12—H12C	109.5
C8—N1—H1A	118.7	H12A—C12—H12C	109.5
C4—N1—H1A	118.7	H12B—C12—H12C	109.5
C20—N2—C16	125.6 (7)	C14—C13—C18	120.5 (8)
C20—N2—H2B	117.2	C14—C13—Br2	118.6 (7)
C16—N2—H2B	117.2	C18—C13—Br2	120.8 (7)
C2—C1—C6	120.1 (8)	C13—C14—C15	119.5 (8)
C2—C1—Br1	119.7 (6)	C13—C14—H14A	120.3
C6—C1—Br1	120.1 (7)	C15—C14—H14A	120.3
C1—C2—C3	119.9 (7)	C14—C15—C16	120.3 (8)
C1—C2—H2A	120.1	C14—C15—H15A	119.8
C3—C2—H2A	120.1	C16—C15—H15A	119.8
C2—C3—C4	120.5 (8)	C17—C16—C15	120.4 (8)
C2—C3—H3A	119.7	C17—C16—N2	119.5 (8)
C4—C3—H3A	119.7	C15—C16—N2	120.1 (8)
C3—C4—C5	120.0 (7)	C16—C17—C18	118.0 (8)
C3—C4—N1	119.9 (7)	C16—C17—C19	121.8 (8)
C5—C4—N1	120.1 (7)	C18—C17—C19	120.1 (9)
C6—C5—C4	117.5 (7)	C13—C18—C17	121.2 (8)
C6—C5—C7	122.0 (8)	C13—C18—H18A	119.4
C4—C5—C7	120.5 (7)	C17—C18—H18A	119.4
C5—C6—C1	121.9 (8)	C17—C19—H19A	109.5
C5—C6—H6A	119.0	C17—C19—H19B	109.5
C1—C6—H6A	119.0	H19A—C19—H19B	109.5
C5—C7—H7A	109.5	C17—C19—H19C	109.5
C5—C7—H7B	109.5	H19A—C19—H19C	109.5
H7A—C7—H7B	109.5	H19B—C19—H19C	109.5
C5—C7—H7C	109.5	O2—C20—N2	120.0 (8)
H7A—C7—H7C	109.5	O2—C20—C21	120.7 (9)
H7B—C7—H7C	109.5	N2—C20—C21	119.2 (9)
O1—C8—N1	120.7 (7)	C22—C21—C24	116.0 (13)
O1—C8—C9	121.6 (7)	C22—C21—C20	114.5 (11)
N1—C8—C9	117.7 (7)	C24—C21—C20	106.8 (12)
C11—C9—C8	109.7 (6)	C22—C21—C23	109.6 (13)
C11—C9—C10	109.6 (8)	C24—C21—C23	98.6 (10)
C8—C9—C10	108.3 (6)	C20—C21—C23	110.2 (10)
C11—C9—C12	110.7 (7)	C21—C22—H22A	109.5
C8—C9—C12	109.9 (7)	C21—C22—H22B	109.5
C10—C9—C12	108.5 (7)	H22A—C22—H22B	109.5
C9—C10—H10A	109.5	C21—C22—H22C	109.5
C9—C10—H10B	109.5	H22A—C22—H22C	109.5
H10A—C10—H10B	109.5	H22B—C22—H22C	109.5
C9—C10—H10C	109.5	C21—C23—H23A	109.5
H10A—C10—H10C	109.5	C21—C23—H23B	109.5
H10B—C10—H10C	109.5	H23A—C23—H23B	109.5
C9—C11—H11A	109.5	C21—C23—H23C	109.5
C9—C11—H11B	109.5	H23A—C23—H23C	109.5
H11A—C11—H11B	109.5	H23B—C23—H23C	109.5
C9—C11—H11C	109.5	C21—C24—H24A	109.5
H11A—C11—H11C	109.5	C21—C24—H24B	109.5
H11B—C11—H11C	109.5	H24A—C24—H24B	109.5
C9—C12—H12A	109.5	C21—C24—H24C	109.5
C9—C12—H12B	109.5	H24A—C24—H24C	109.5
H12A—C12—H12B	109.5	H24B—C24—H24C	109.5

### Hydrogen-bond geometry (Å, °)

**Table:**

*D*—H···*A*	*D*—H	H···*A*	*D*···*A*	*D*—H···*A*
N1—H1A···O2^i^	0.86	2.14	2.989 (8)	170
N2—H2B···O1^ii^	0.86	2.14	2.943 (8)	155

Symmetry codes: (i) −*x*+1, −*y*, −*z*+1; (ii) *x*, −*y*+1/2, *z*−1/2.
